# Minimal Linear Networks for Magnetic Resonance Image Reconstruction

**DOI:** 10.1038/s41598-019-55763-x

**Published:** 2019-12-20

**Authors:** Gilad Liberman, Benedikt A. Poser

**Affiliations:** Faculty of Psychology and Neuroscience and Maastricht Brain Imaging Center, Maastricht University, Maastricht, The Netherlands

**Keywords:** Magnetic resonance imaging, Applied mathematics

## Abstract

Modern sequences for Magnetic Resonance Imaging (MRI) trade off scan time with computational challenges, resulting in ill-posed inverse problems and the requirement to account for more elaborated signal models. Various deep learning techniques have shown potential for image reconstruction from reduced data, outperforming compressed sensing, dictionary learning and other advanced techniques based on regularization, by characterization of the image manifold. In this work we suggest a framework for reducing a “neural” network to the bare minimum required by the MR physics, reducing the network depth and removing all non-linearities. The networks performed well both on benchmark simulated data and on arterial spin labeling perfusion imaging, showing clear images while preserving sensitivity to the minute signal changes. The results indicate that the deep learning framework plays a major role in MR image reconstruction, and suggest a concrete approach for probing into the contribution of additional elements.

## Introduction

Deep learning (DL) has been playing a significant role in many fields of science, including imaging and medical image processing. Recently, DL techniques have also been applied to medical image reconstruction^1–4^. It has been shown that DL approaches can outperform state-of-the-art compressed sensing (CS)^5^ techniques to translate the signal acquired by an imaging device into a usable medical image by means of a suitable domain transformation.

In this work we introduce a minimal linear network (MLN) for magnetic resonance (MR) image reconstruction capable of outperforming best available CS and dictionary learning alternatives, and show its applicability under benchmark simulation tests and challenging imaging conditions, where near artefact-free images were obtained. We emphasize model simplicity, allowing us to probe into the elements that contribute to the recent successes of DL-based MR image reconstruction.

DL approaches to MR image reconstruction that have been investigated to date include: (a) using Neural Networks (NN) to improve images post-hoc after standard reconstruction^1^; (b) reconstructing from the signal domain (*k*-space) by Fourier transformation (FT)^2^, typically in combination with multi-coil parallel imaging reconstruction^6,7^; (c) training an NN for the full transformation from signal into image domain through a representation manifold^4^; (d) mimicking CS iterative reconstruction techniques while allowing more versatility through non-linear operations^3,8,9^, following the assumption that MR images lie in a restricted manifold and using ideas from DL as learnt representation features of the data from training samples.

Linear neural networks have been previously analyzed^10^, and were shown to converge in a manner similar to non-linear networks^11^, in general domains. Nonlinear activation functions for Neural Networks on the complex field^12^ have been applied to e.g. MR fingerprinting^13^ reconstruction^14^.

Of important note is that MRI differs fundamentally from other imaging modalities: images are inherently complex, with important information contained in the phase; they are acquired indirectly by sampling a different domain (spatial frequency i.e. Fourier domain known as *k*-space); they are multidimensional, with different axes being encoded in a different manner. Commonly, a readout (RO) axis acquired continuously at high bandwidth, and phase-encoding (PE) axis acquired step-wise at low bandwidth (both in the spatial frequency domain), and a slice-select (SS) axis, acquired directly in the image domain by slice-selective excitation. Further dimensions/axes include multiple receive channels (for parallel imaging), timeseries or any other data dimension that varies during the scan (e.g. diffusion weighting or direction).

Conventional MRI reconstruction thus commonly consists of a series of linear transforms. In the simple case of fully sampled, single-channel *k*-space data, this involves FT on the RO axis, followed by FT on the PE axis. With modern vastly parallel reception, software coil-compression^15^ is often performed to reduce computation load, by multiplying the data on the channel dimension by a coil-compression matrix. These operations are matrix multiplications on one axis, while the weights are shared (for coil-compression, FT) or independent (for channel combination) on the other axes. Axes-specific tensor dot product generalizations are ubiquitous in MR image reconstruction.

The workhorses of neural networks are the fully-connected (FC) and convolutional layers. FC layers connect all neurons from one layer to the next, losing all localization information and enabling domain transformation (e.g. from spatial frequency to image space). Convolution layers, by contrast, have a neighborhood connectivity topology that preserves location, and the weights of the kernel are shared among all instances along the image.

Recently, separable convolutions, i.e. structured nD convolution operation which is separable to several convolution operations of lower dimensionality, have been successfully introduced into neural networks applications^16,17^. In this work, a similar concept is suggested for separable nD tensor multiplications, i.e. for axes-dependent FC layers.

NN in application to MR image reconstruction benefit from a variety of recent advances such as non-linear activation layers, deep topology, practically efficient algorithms and powerful parallel computing technology, and availability of large training datasets. Major disadvantages, however, are the lack of interpretability, and the lack of consistent procedures for designing optimal/minimal topology and understanding of elements’ contribution. Therefore, despite great successes, the ambiguity and inconsistency in method selection, understanding the results and what has contributed to a network’s success have raised concerns that may hold up widespread acceptance into routine use. This calls for a different approach to neural networks that allows for the interpretation of results, the design of minimal models to achieve the goal, and careful assessment of each element’s contribution. Implementation of NN that obey these principles is the core aim of this paper.

Following this direction with focus on MR image reconstruction, we restrict the present work to linear networks/models, thereby shifting the focus from learning local features to robust inversion of subject-specific ill-conditioned systems. Thus, no activation layers are used and bias weights are discarded throughout. The proposed models and layers operate on the complex field. These ill-conditioned linear systems are however subject specific.

This results in an interpretation of the recently suggested AUTOMAP^4^ as a GRAPPA-type reconstruction technique. We thus suggest a way to reduce it to its essential elements, improving performance and interpretability. This can be viewed as a model-based GRAPPA, which is also a generalization of GRAPPA to arbitrary non Cartesian trajectories.

We are further motivated to test this approach in a challenging real-world MR image reconstruction setting, of an *in-vivo* perfusion experiment. In this scenario, limits to acquisition time result in the need for higher acceleration rates. Along with the influence of B_0_ field-inhomogeneities at ultra-high field (7 Tesla), this results in a challenge in which current state-of-the-art approaches fail to deliver usable images.

We introduce a family of connection layers fit for use in axis-dependent problems such as MR image reconstruction, and propose a scheme of *minimal* linear networks (MLN), that are minimal in topology and in the number of weights to tune, allowing them to benefit from pulling more weight sharing, simpler models, and smaller computational/memory demands, while making them more interpretable analogous to conventional reconstructions.

We demonstrate that learning from large realistic datasets, using the common advanced backpropagation algorithms, is by itself very powerful, and should be explored separately from other neural network elements (e.g. deep networks, nonlinear activations and redundancy of features).

We conclude by applying the proposed schemes to the problem of undersampled spiral acquisitions at ultra-high field, and demonstrate its application to a notoriously low-SNR, Arterial Spin Labeling (ASL) *in vivo* perfusion experiment^18,19^. We thus demonstrate the existence of a instance-specific matrix that is a robust left-inverse for the 7 T undersampled spiral trajectory system, with domain of outperforming state-of-the-art algorithms which include the images scanned.

### Connection layers

Table 1 summarizes previously and proposed connection layers. Current NN architectures are dominated by convolution layers and fully connected (FC) layers. Locally connected layers^20,21^ have been suggested, but not yet widely explored.

**Table 1:** Currently used connection layers (marked with*) and the suggested ones.

Weights between instances→		Shared	Independent
Topology↓			
Subspace Fully connected (SFC)	All axes fully connected*	Only one set of weights*	
		SFCs	SFCi
		SFCg	
	0-axes fully connected/1x1 kernel neighborhood network	linear combination of features	Maps
Neighborhood		Convolution layer*	Locally-connected layer*^20,21^
		Locally Connected Layer with axis-dependent weight sharing	

An FC layer connects all nodes from one layer to the next. In a convolution layer, each neighborhood of “neurons” in one layer is connected to a respective neuron in the next layer, and the weights are shared between instances of that connection (i.e. per neuron in the receiving layer). Thus, FC layers and convolution layers differ in two aspects: connection topology, and weight sharing. Table 1 is organized according to these two aspects. Locally connected layers have the topology of convolutional layers, but not the weight sharing. An FC layer is a matrix multiplication, where each row corresponds to the weights connecting all neurons of one layer to a specific neuron in the next layer. For illustration, consider a layer with MxNxF_1_ neurons (e.g. describing an MxN image with F_1_ features) connected to the next layer, of size MxNxF_2_. An FC layer will be composed of M^2^⋅N^2^⋅F_1_⋅F_2_ weight variables (+M⋅N⋅F_2_ additive bias variables). A convolutional layer, with K_1_ × K_2_ kernel, will have K_1_⋅K_2_⋅F_1_⋅F_2_ weight variables (+F_2_ bias variables as they are fully connected in the feature dimension). The neighborhood topology helps by limiting the number of variables to optimize, while the shared weights serve to reduce the number of variables to optimize, and to strengthen each optimization step by combining the information from all the data patches.

### Axis dependent topology and weight sharing

**Figure 1:**
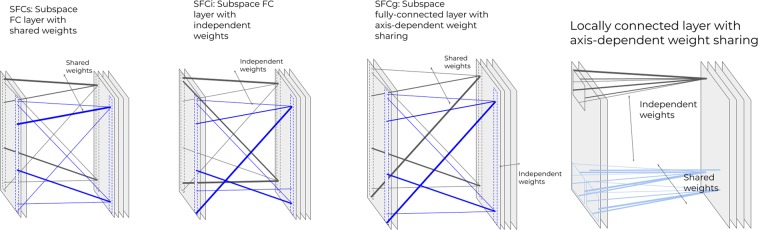
Layers proposed in this work.

A 1D FT of a tensor, for example, may be described as a layer with specific topology such that it is fully-connected on one axis, while the weights are shared along the other axes. Thus, we suggest a *subspace-connection* layer, where some axes are fully connected, the weights shared along some of the other axes and independent along the remaining axes. Next, we demonstrate the applicability of this layer to common MR reconstruction schemes. The proposed layers are depicted in Fig. 1.

### Notation

In the remainder of this paper we will use the following notation to denote use of the proposed connection layers: [Fully connected: Source- > New | Shared | Independent]. In the fully connected case, location information is lost, and sometimes a domain change (e.g. spectral- > spatial: *k*-space to image space) is indicated. The names of the source and new axis (e.g. *k*_RO_- > RO) is noted for clarity.

## Results

### 2D-FT and 2 × 1D-FT

As a first step, we compared two networks performing 2D FT, one using a 2D-FC single layer and one composed of two FC-1D layers with weights sharing in the other dimension (see Supplementary Fig. S1). For an NxN image, the number of weights is the two networks is thus N^4^ and 2N^2^, respectively. The 2 × 1D network showed more rapid convergence (shown in Supplementary Fig. S1C). For the N = 128 case, the 2D-FC network did not fit into the device memory. Notably, for the case of N = 64 and a fast learning rate of 0.002, training the 2D-FC topology did not converge, while the 2 × 1D network was robust to this rate.

### Coil compression

**Figure 2:**
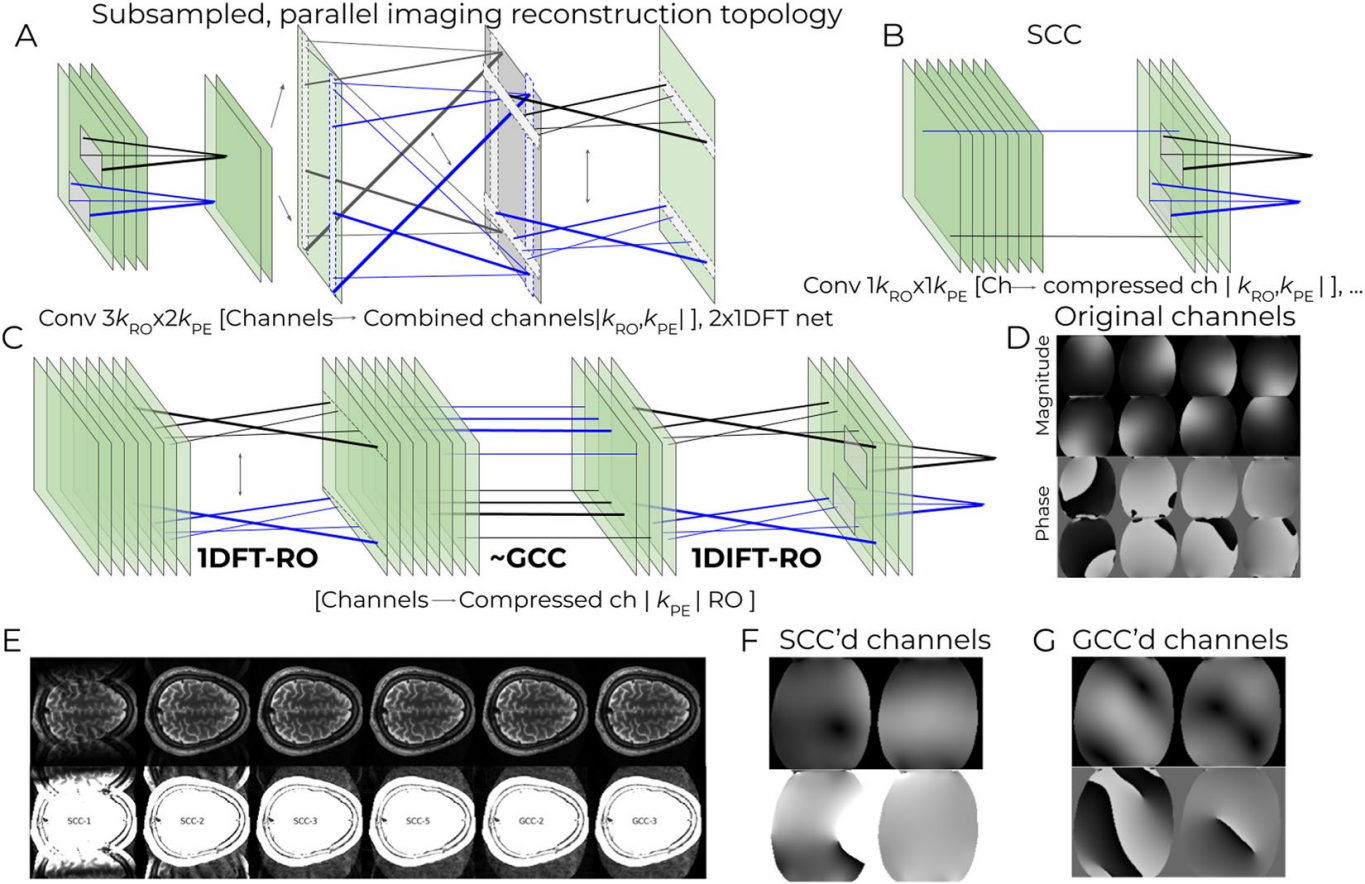
(**A**) Basic subsampled, parallel imaging reconstruction network. (**B**) Software coil compression (SCC) and (**C**) geometric coil compression (GCC) modules that can be inserted before the network depicted in A. (**D**) Sensitivity maps of an eight-channel coil used in the simulations. (**E**) Results of image reconstruction using various SCC and GCC settings, with the number of target coils indicated in the lower row with reduced dynamic range emphasizing ghost artifacts. (**F**,**G**) show the sensitivity maps of the two coils resulting from training the SCC- and GCC compressed parallel-imaging reconstruction networks, respectively.

Multi-channel data that are undersampled along the phase-encoding direction can be reconstructed by applying a convolution kernel shared over *k-*space and FC over the channel dimension to fill-in the missing lines, followed by a 2D (or 2 × 1D) FT. The kernel weights are usually calculated to fit the auto-calibration lines, or by the coil sensitivity maps from a separate acquisition. A network mimicking this reconstruction pipeline is depicted in Fig. 2A. (This toy example borrows elements from GRAPPA^7^ as the k_x_-k_y_ block, and elements from SMASH^22^ as the single combined output channel). Training this network resulted in extraction of a kernel which enabled successfully reconstructing images from undersampled data.

### Software Coil Compression(SCC) and Geometrical Coil Compression (GCC)

Coil compression is commonly applied as a first step in parallel imaging reconstruction to decrease computational demands^15,23–25^ and reduce noise. A network element capable of performing software coil compression is depicted in Fig. 2B. Compressing the example data set from eight to two channels resulted in compressed channels with expected phase variation along the PE dimension (Fig. 2F), allowing for parallel imaging reconstruction.

A network mimicking Geometrical coil compression^25^ is depicted in Fig. 2C. GCC achieves more efficient channel reduction by using aligned spatial-location-specific coil compression, and is thus built up using 3 SFCg layers: (i) performing FT: shared weights along PE, channels, matrix multiplication along *k*RO- > RO axis, before FT back to *k*-space. In the network implementation, (ii) location-specific coil compression: independent along RO, FC along channels- > compressed channels; shared along *k*PE; (iii) performing iFT, as (i). Step (ii), the core of GCC, thus exemplifies a common use of the full versatility of subspace FC operation. As illustrated in Fig. 2C, weights (different line widths in the figure) are different along one axis, and shared along the other axis (i.e. the black and blue). Matrix multiplication operation is run on the feature (channels- > compressed channels) axes.

Unlike the analytical implementation of GCC, the network needed no explicit constraint to be added for coil compression matrices to align and produce smooth compressed channels. The defined goal of reconstructing a correct image is sufficient to constrain the network to produce the desired behavior, i.e. smoothness of the resulting compressed channels. Figure 2E shows resulting image reconstructions, showing the network was able to learn more effective compression (than SCC) due to the added GCC flexibility. Figure 2G shows the resulting compressed coils, demonstrating the smoothness achieved without explicit constraints.

### Reconstruction from spiral trajectory acquisition

Spiral trajectories enable high-efficiency coverage of *k*-space. However the spiral readout results in data points not lying on the Cartesian grid, requiring alternative approaches such as location specific regridding kernels^26^ or NUFT^27–29^ for image reconstruction. Here we explore the use of MLN for the reconstruction of such trajectory under field inhomogeneity common in 7 T systems.

### MLN for spiral reconstruction

**Figure 3:**
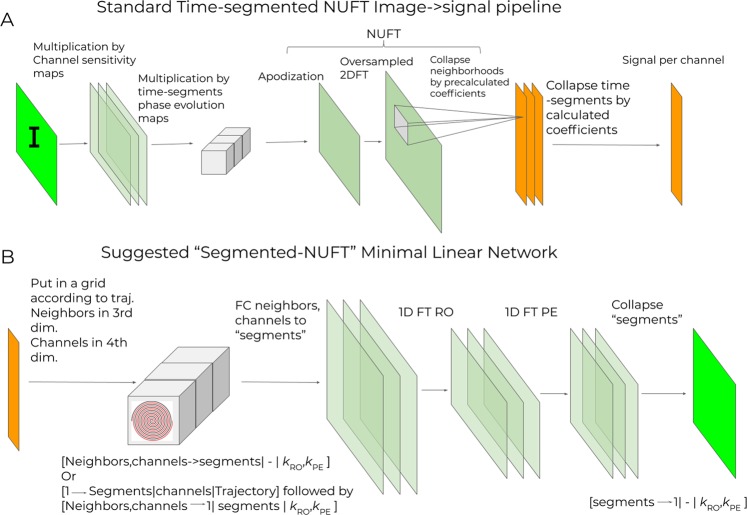
(**A**) Standard time-segmented^28^ image-to-signal operator for use in iterative reconstruction from non-Cartesian sampling under field inhomogeneity. (**B**) Corresponding Minimal Linear Network with *k*-domain location specific regridding kernels into “time-segments”.

Figure 3A shows a standard time-segmented^28^ image-to-signal operator for iterative reconstruction from non-Cartesian sampling under field inhomogeneity. Two topologies of Minimal Linear Networks were designed: one tightly mimicking the transposed time-segmented NUFT pipeline, and another slightly more relaxed version, composed of a *k*-domain regridding layer into several “time segments” (TS), a fixed 2D FT and an image-domain side combining the segments into the final image. We will refer to the latter topology as *k* + I MLN.

In the first topology, N_Traj_⋅N_segments_ + N^2^⋅N_Neighbors_⋅N_Channels_ + N^2^⋅N_Segments_ weights are trained by the network, equal to the number of coefficients calculated by a time-segmented NUFT operator, where N_segments_ is the number of time-segments.

The *k* + I topology, depicted in Fig. 3B, has N^2^⋅N_PE_⋅N_Neighbors_⋅N_Channelsj_⋅N_segments_ + N^2^⋅N_Segments_ weights trained by the network. Table 2 compares the resulting number of tunable weights in a specific configuration. In contradiction with linear networks, nonlinear networks are universal approximators^30,31^, and the expressiveness of the network grows exponentially with depth^32,33^. Thus we keep the comparison to comparable topologies. While the number of tunable parameters in MLN is still high compressed to e.g. convolutional networks, the linearity of MLN means the system cannot do anything but projection of the input into a vector space, and only vector spaces can be learned.

**Table 2:** Number of trainable weights in different topologies.

	#Tunable weights N = 128 matrix size, N_ch_ = 13 channels, N_TS_ = 7 Time segments, N_N_ = 12 Neighbors, Acc = 4	
AUTOMAP^4^	1,409,286,144	N_ch_⋅N^4^/Acc + 2⋅N^4^ + C
Full general-purpose linear transform	1,744,830,464	2N_ch_⋅N^4^/Acc
*k* + I MLN, TS independent	36,012,032	2N_ch_⋅N_Segments_⋅N_N_⋅N^2^ + 2N^2^⋅N_TS_
MLN, TS shared	5,398,528	2⋅N^2^⋅N_ch_/Acc + 2N^2^⋅N_Segments_⋅N_N_ + 2N^2^⋅N_TS_
MLN, no B_0_ correction	5,111,808	2⋅N^2^⋅N_ch_⋅N_N_

The full general-purpose linear transform transforms is similar to the main layer of AUTOMAP - transforming directly from signal data to reconstructed image.

The recently suggested dAUTOMAP^34^ achieved results comparable to AUTOMAP while much reducing the number of parameters by decomposition, not dissimilar to the one approach taken in this work. However, it does not fit non-Cartesian trajectories.

Both topologies were also ran with a single segment, mimicking NUFT without B_0_ inhomogeneity correction. Note that with N_Segments_ = 1, both topologies became nearly identical.

**Figure 4:**
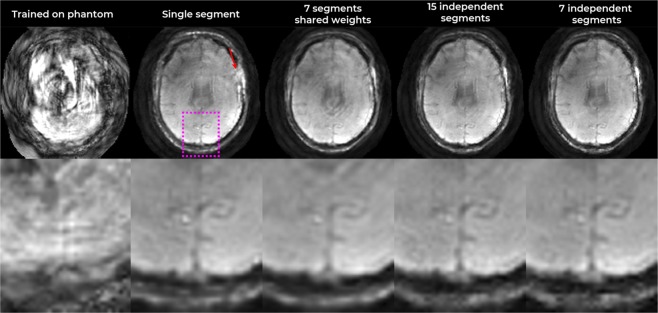
Top row: Result of image reconstruction after training the network using multi-ellipse phantoms only. Bottom row: Image reconstruction after training various network topologies. The bottom row shows zoomed views. Red arrow indicates a region with strong field-inhomogeneity induced artifact.

Figure 4B shows the reconstruction with the first topology and N_Segments_ = 7, and with the second topology and N_Segments_ = 1,7,15 segments. The number of segments is in the order of the number of time-segments needed for sufficiently accurate description of the phase evolution due to field inhomogeneity. As expected, the single-segment (non-B_0_ corrected) reconstruction showed distortion and blurring in regions of strong inhomogeneity (marked by red arrow). The time-segmented reconstruction with shared-weights resulted in an image with some residual blurring. Comparing the reconstructions with 7 and 15 independent-weights segments did not indicate further gains when using > 7 segments and the variant with 7 independent weights (i.e. ~2.1 ms difference between segments, see Methods) was hence chosen for further exploration.

The *k* + I MLN was also trained using a database of simulated multi-ellipse (modified Shepp-Logan) phantoms. The result of then applying this to reconstruct the actual *in vivo* data is shown in Fig. 4, clearly illustrating that the phantom-trained network did not generalize to the real data, indicating that the real data (as MR brain images) do not lie within the vector-space (or, have a high cost in the linear scalar field) learned by the MLN using multi-ellipses. It did perform well in reconstructing similar multi-ellipse phantom data (not shown).

### Comparison with reference reconstruction techniques

**Figure 5:**
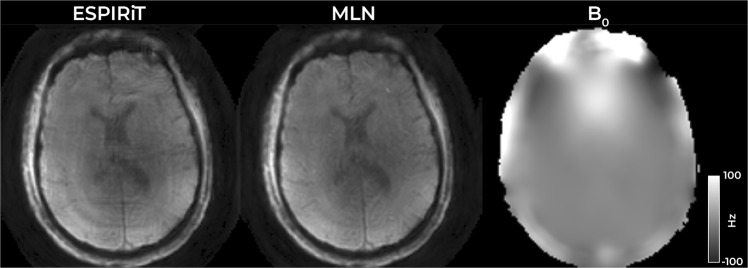
Result of image reconstruction from real data using state-of-the-art regularized CS technique and the proposed method, as well as the measured field inhomogeneity.

Figure 5 summarizes the comparison to established regularized CS-based reconstruction technique. Suggestions for direct regridding through interpolation kernels^27^ include optimized Kaiser-Bessel, Gaussian, minimal least squares and other approaches. However, the state-of-the-art methods for non-Cartesian image reconstruction combine CS with kernel interpolation for the image-to-signal operations. We have tested BART^35^ as reference reconstruction (relying on Kaiser-Bessel kernels, and Toeplitz embedding). SparseMRI^5^ with optimized Keiser-Bessel showed comparable results (not shown). Supplementary Dataset 1 contains more complete comparison with reference reconstructions, for all of the subjects and slices scanned.

The results reveal artifacts in the reference reconstructions, while the proposed MLN produces a clean and detailed image of improved quality. The consistency of the results over all scanned subjects is summarized in supplementary dataset SD1A and detailed in supplementary material SD1B.

### Application to perfusion measurement with arterial spin labelling at 7T

**Figure 6:**
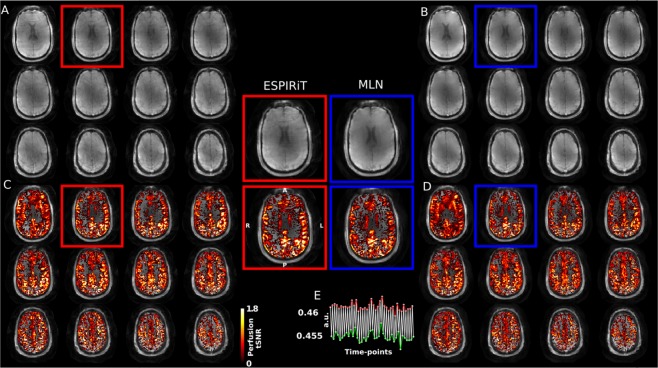
Perfusion experiment at 7 Tesla: Result of multi-slice image reconstruction from actual spiral MRI acquisitions using (**A**) a regularized CS-based technique (**A**) and the proposed method (**B**). Panels (C,D) show the corresponding tSNR maps. The center part shows zoomed-in versions of one selected slice. (**E**) Shows the ASL signal time course of the perfused region of interest where green and red colours indicate the label and control time points respectively.

Due to the linearity of the model, we expect it to generalize well. The temporal variations between the label and control images in an Arterial Spin Labelling (ASL) experiment are notoriously low, around 1%^36^. The results shown in Fig. 6 demonstrate the method’s sensitivity to these minute changes and are in good agreement with conventionally obtained perfusion results using echo-planar ASL at 7 T^19^. Figure 6E shows the time course signal average over the activated area. Indeed, a 1% signal change was detected, indicating the full difference between labeled and unlabeled timepoints was preserved. While tSNR values are slightly lower using MLN than by the reference reconstruction, the maps are cleaner and fuller, without loss of contrast in areas due to artifacts and regularization.

### Interpretability of MLN

**Figure 7:**
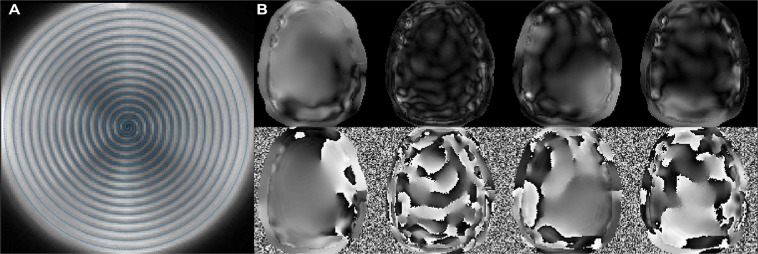
Interpretability of the proposed MLN. (**A**) shows the root-mean-square (rms) value of regridding kernel weights per pixel, with the sampled trajectory overlayed in blue. Grid locations closer to acquired points have lower rms weight. (**B**) Magnitude (top) and phase (bottom) of four of the “segment” maps, extracted from the weights of the trained network’s last layer.

The chosen network is composed of trainable weights before and after FT. Figure 7A shows the Frobenius norm of the signal (*k*) side weight matrix for each *k*-space location. As expected, the locations which lie on the sampled trajectory enjoy a small norm, while those between sampled locations demand higher weights, indicating stronger use of the different channels’ and neighbors’ data, and increased noise for these locations. In the image domain, the trained weights can be depicted as maps, and are shown in Fig. 7B. The trained maps contain features that are reminiscent of the field inhomogeneity (B_0_) map, in similarity with time-segmentation maps, but also of compressed channel sensitivities.

### Benchmark performance in Cartesian reconstruction

**Figure 8:**
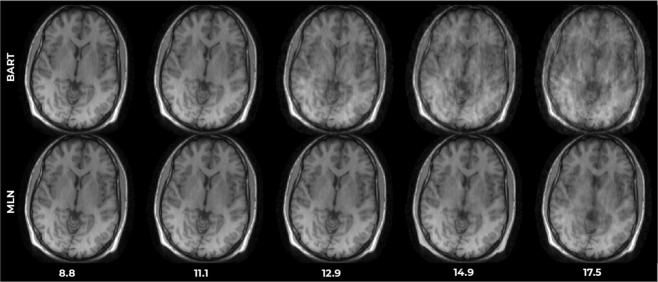
Results of image reconstruction from eight-channel Cartesian data with various undersampling factors, using the proposed (MLN) and reference (BART) methods.

The *k* + I MLN topology was applied to image reconstruction from standard 2D Cartesian 8-channel example data that was undersampled post-hoc. The SSIM score^37^ was used as a quantitative metric of image quality. Figure 8 shows the performance at different undersampling factors on a standard image not within the training database. In each case, the MLN reconstruction outperforms the reference method, notably producing images with acceptable quality even at factor > 12. Fig. S2 shows the performance on additional test-set images which do not belong to the training database, including complex-valued images acquired at our institution, with SSIM scores summarized in Table S1.

Similar trends are seen for newly acquired complex-valued 7 T MR images, publicly available magnitude MR images, simulated phantom images and even general non-MR images. The proposed MLN reconstructions outperformed the reference method in all cases, indicating that the MLN learned a vector subspace (linear scalar field) more general than the training samples brain MR images. This follows the relatively weak descriptive power of the architecture, which restricts in to linear relations, and enjoys generalizability and reduced risk of overfitting.

### Comparison to neural network

**Figure 9:**
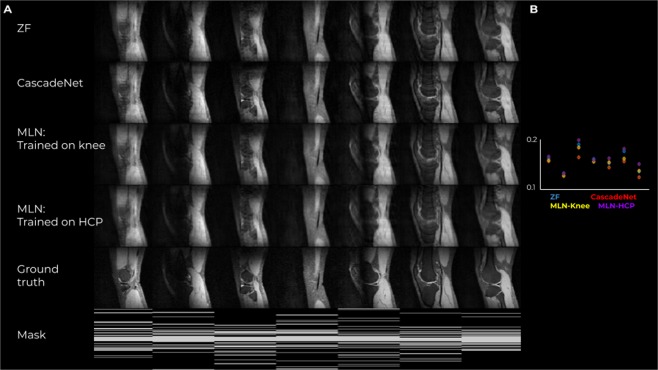
Results of image reconstruction from single-channel Cartesian data with 1D Poisson masking, using the proposed (MLN) and CascadeNet. (**A**) Reconstructed images and sampling masks. (**B**) NRMSE values.

Figure 9 Shows results in comparison to the CascadeNet on single-channel data. Notably, CascadeNet outperformed MLN, and MLN-Knee outperformed MLN-HCP. In this experiment, networks are challenged to extract information on the image manifold from the database, rather than utilize a coil’s channels. As MLN was designed to have a low, linear descriptive power, its ability to reconstruct the data in such settings are expectedly low. The gap between the MLNs trained on Knee and HCP data reflects that those datasets lie in a different vector spaces. The results thus confirm the observation regarding the limited model used by MLN, which is aimed to mimic a linear GRAPPA operation using model-based training on arbitrary trajectories.

### Noise considerations

Fig. S3 shows the noise calculated using the proposed MLN and reference method on simulated data, and Fig. S4 on real 7 T MRI *in-vivo* acquisitions. In both cases the MLN reconstruction suffered from increased noise compared to the reference method, however with smaller difference in the real data. As the trained MLN does not contain any element effectively applying denoising, such as conv-net, or the regularization used in the reference method, some noise amplification is expected. Several approaches to reduce the noise amplification include the introduction of nonlinear, effectively denoising layers; weights regularization; denoising the training images; and adding realistic noise to the simulated signals.

## Discussion

Deep learning brings various benefits over conventional approaches to image reconstruction. Among these are: (a) efficient use of computational resources; (b) learning from large databases; (c) optimizing directly in the result domain; (d) exploiting redundancy for robustness and over-complete representations, and (e) learning nonlinear functions. However, indications for the construction of a successful network remain vague with commonly used general topologies. Evidence presented in this work indicates that even when removing the hallmark elements of neural networks, namely non-linear activation, deep topology and redundancy (overrepresentation), elements a-c alone already outperforms state-of-the-art techniques. Lacking the non-linear activation layers, the resemblance to “neurons” diminishes, and we hence use the term linear nets, or “minimal linear nets”, MLN. A concrete criterion for minimality is the inability of any reduced model to fully describe the underlying physics.

We propose connection layers that fit MR and other axis-dependent domains and applied them to design minimal, problem-specific and physics-based networks for the various elements in a typical MR reconstruction pipeline. This enables analysis of each connection layer’s role. The use of minimal architectures is analogous to the traditional concepts of model regularization for improved generalization.

Instead of learning features and patterns of the possible image space, the suggested MLN suffice in learning the signal-image relation in the restricted “possible image” vector space, thus decreasing the complexity learned and embedded into the system. As NN reconstruction trains a signal-to-image function by iteratively augmenting incorrect reconstruction of the training set, we explore the power of this element without adding powerful image-based pattern description mechanism.

In application to the real-world example of 7T spiral ASL perfusion measurements, we demonstrated the method’s robustness under challenging imaging conditions, and applicability to timeseries imaging.

The robustness of MLN compared to conventional reconstruction approaches can be explained by adopting an interpretation by which the learned network is a robust inversion function that is trained directly as a left-inverse, and thus emphasizes *correct* rather than “*good*” reconstruction.

Treating the MR reconstructions with a problem-specific architecture resulted in orders-of-magnitude decrease (~500×) in the computational and memory demands in comparison with the general signal-to-image transform, and allowed reconstruction on a commercial desktop computer in cases where the computational demands of a general approach are too high.

This work focused on inverting fully known but underdetermined linear systems. Thus, and also for clarity of presentation, only pure linear networks were used. However, another domain of MR reconstruction challenges deals with problems where the forward image-to-signal function is not fully known: For instance, in cases where motion or changes in the B_0_ field affect the image. In this case, use of common neural network techniques as non-linear activation functions in combination with redundancy is expected to be beneficial; but even then, MR physics will allow us to constrict the expected artifact, for example, to the low-bandwidth (phase-encoding) axis. In such cases, we propose the use of the connection layers introduced here, in combination with problem-specific nonlinear layers, which we will explore in future work.

For real MRI experiments, the choice of a relatively difficult imaging scenario which demands estimation and accounting for the sensitivity maps and the B_0_ inhomogeneity, required us to generate training data that is subject and slice specific, resulting in high computational, time-consuming demands. In order to achieve real-time or near real-time reconstruction, a network should be designed with robustness to changes in these properties. In such cases, the inverse, signal-to-image function is highly non-linear and cannot be described using linear models, which will hence require some degree of nonlinearity to be introduced to the system.

In conclusion, this work in the context of MR image reconstruction indicates a greater role for the learning from training sets and utilization of the slow-learning back-propagation optimization algorithm, than advanced description of the MR image manifold.

## Methods

### Training dataset

For training, 10,000 magnitude-only images were randomly chosen from the Human Connectome Project^38^ database. The images were randomly taken from axial, sagittal and coronal reslicing, and were resized into a 256 × 256 matrix. Data augmentation included random cropping to 128 × 128, flipping in both dimensions and 90° rotation. A random phase was added, generated using the sum of sinusoidal functions over a randomly selected quadratic plane.

### Spiral acquisitions at 7T

Experiments were performed on four healthy volunteers (24–39 years old, 1 female) after obtaining informed consent. The study was approved by the Ethics Review Committee for Psychology and Neuroscience (ERCPN #180_03_06_2017) at Maastricht University and all procedures followed the principles expressed in the Declaration of Helsinki. Data was acquired on a Magnetom 7 T whole-body MRI research scanner (70 mT/m amplitude, 200 T/m/s slew rate gradients; Siemens Healthineers, Erlangen, Germany), with a 32-channel receive head coil (Nova Medical Inc, Wilmington, MA).

At ultra-high field, B_0_ field inhomogeneity poses significant challenges to fast echo-train imaging such as EPI or spiral trajectories. Moreover, the short T_2_* requires use of short TE in some applications which at the desired resolution, however, may be precluded by the echo-planar readout duration. ASL for perfusion imaging is one method that benefits greatly from shorter echo-time, making a spiral-out readout an attractive solution. An ASL sequence with spiral readout was developed in-house, with the FAIR^39^ QUIPSS II^40^ labeling scheme with tr-FOCI inversion pulse^41^ to obtain quantitative maps of perfusion using a single subtraction approach^42,43^. All ASL measurements followed^19^, i.e. had 12 slices with no interslice gap, FOV 192 mm, echo time (TE)/TI1/TI2/TR1 = 3/700/1,800/2,500 ms, 67°−90° excitation flip angle (according to each subject’s Specific Absorption Rate limits), 80 repetitions, total scantime 4 minutes. Dielectric pads were used to improve labelling efficiency^44^. Variable-density spiral trajectories were designed according to^45^, duration 12.5 ms, with effective undersampling factor of ~3.2.

Multi-echo GRE data (TEs 1.5, 3.18 ms) with matched geometry was acquired separately for B_0_ field mapping and coil sensitivity estimation.

### Network topologies used for Spiral reconstruction

Two topologies were designed: (i) A minimal linear network (MLN) topology tightly mimicking the transposed time-segmented NUFT pipeline: the trajectory data is first expanded by the number of “segments”, using kernels that are trajectory location specific, but shared along channels. These are then rearranged into a grid, with each grid location containing a concatenation of the data from neighboring *k*-space locations on the trajectory, and the different channels, along a new channel/neighbor dimension. Those are collapsed into N_segments_ value using learned location specific kernels (shared along segments). The data are then Fourier transformed (using a fixed, non-trained standard FT matrix), and the resulting images from the different segments are collapsed into the final reconstructed image.

(ii) relaxed variant, depicted in Fig. 3B: the data are first arranged into an oversampled *k*-space grid, collecting data from neighboring *k*-space locations and channels along a new dimension, which is reduced to length N_Segments_ using *k*-domain specific kernels, followed by the rest of the network as before.

The networks, specific for subject and slice, were trained to reconstruct images from simulated signal. Signal was simulated from *database* images (HCP, with data augmentation as detailed above), with slice-specific sensitivity and B_0_ maps, obtained in a separate acquisition on the subject, and with the nominal trajectory, according to the image-to-signal transform suggest in^28,46^.

### Benchmarking

The MLN was trained using the same training samples on 8-channel sensitivity maps (shown in Fig. 2B). Poisson disk undersampling masks were computed using the “poisson” module of BART at various (equal) accelerations in both axes.

For comparison of the reconstruction with BART *pics* module, it was run with wavelet regularization, with weights of 10 to the power of -8, -7, -6, -5, -4, -3.5, -3, -2.5, -2, -1.5, -1; for each image and each acceleration factor, the one that gave the highest SSIM was chosen.

Dictionary learning was run with recommended parameters (98 patches of size 7 × 7; lambda = 140 and thresholds as in the provided code). Iterations were run until relative change in reconstructed image energy went below 7e-3, which provided good reconstructions in accordance with published results.

Test set: the reconstruction was tested on an independent set of images from several categories: a standard test brain image (taken from the ESPIRiT publically available demo code); magnitude abdomen MR images from the public domain (https://images.computerhistory.org/makesoftware/5.6_Abdominal-MRI.jpg); a natural magnitude image of a house; a multi-ellipse phantom image; and two complex-valued brain images acquired in our institution.

Additionally, MLN were compared to CascadeNet^47^, based on single-channel Cartesian data, in order to match the provided implementation. 2 MLNs were tested, with training on HCP data, as described above, and on the knee data, as the one used by the CascadeNet. NRMSE values are reported.

### Implementation

The networks were implemented in TensorFlow using a standard configuration (Adam optimizer with fixed learning rate (unless mentioned otherwise) of 0.002 and beta = 0.9, L_1_ loss) on a desktop workstation with commercial GPU (NVIDIA GTX 1080 Ti with 11GB of memory). The networks were trained by applying the slice-specific B_0_ field map and sensitivity maps.

Networks were implemented to perform complex-field operations. No activation layers were used. B_0_ maps were calculated using the method of Cusack *et al*.^48^. Coil sensitivity maps were calculated with BART’s ESPIRiT module^49^ using default parameters, and software coil compression (SCC) of the experimental MRI data was done using SVD. For reference reconstruction, BART’s *pics* module^35^ with manually optimized parameters was expanded with implementation accounting for B_0_ correction with time-segmentation^28^, using up to 15 time segments, equally spaced over the acquisition time. NUFT^50^ was also calculated using gpuNUFFT^51^. The images were also reconstructed using either the sparseMRI package^5^, or L_1_-CG-ESPIRiT with L_1_-wavelet or split TV and L_1_-wavelet regularization^49^. Parameters were set manually to optimize image quality.

The ASL data was motion corrected using ANTs^52^ and brain extracted using FSL BET^53^ following which perfusion tSNR maps were computed.

### Memory and performance considerations

The memory requirements for training are linear with the size of the two tensors calculated (the k- and I- side tensors). In these experiments, we also loaded the database to the GPU memory for increased performance. For the real-data spiral trajectories, training was run for 2 hours per instance, which achieved convergence. Inference was done by directly applying the calculated kernels.

### NUFFT for MRI under field inhomogeneities

For the sake of completeness, we briefly summarize the approach developed in^27,28,46,50^ for MR image-to-signal transform under field inhomogeneities and its use for image reconstruction, which was used for reference reconstruction as detailed above and laid the basis for the suggested networks’ topologies.

Let *I* be an image, and *F*_*u*_ be the non-uniform Fourier transform according to the given trajectory, acquiring N data points in acquisition duration *T*. Set *L* to be a (small) number of “time-segments”, separated by duration $\Delta =T{/}L$. The local phase evolution due to the field inhomogeneity at time-segment *l*, i.e. after *l*Δ acquisition time units, is ${P}_{l}(r)={e}^{-i{B}_{0}(r)l\Delta }$, and the image affected by the phase evolution is ${D}_{l}=I\odot {P}_{l}$, where $\odot$ marks element-wise Hadamard multiplication. Let *G* be an *N*x(*L + 1)* matrix containing interpolation coefficients for each (discrete) timepoint (e.g. linear of Hanning interpolation, or interpolation matrix optimized for the specific case, such as via the min-max approach^28^. The signal at time t can be approximated by $\hat{s}=\mathop{\sum }\limits_{j=0}^{L}{F}_{u}\{I\odot {P}_{j}\}\odot {G}_{j}.$ A NUFFT operation can be effectively and accurately approximated by (sparse) matrix multiplication of the FFT of the image with a precalculated coefficients matrix *C*, which is non-zero for a row corresponding to spatial frequencies *f*_*x*_*,f*_*y*_ only in a window around those frequencies^50^.

Conversely, The input to the network is the data, put into the 4-dimensional tensor S, of the form, [kRO,kPE, Neighbor, Channel]. That is, at each kx,ky location, the data from the NNeighbors nearby (non-Cartesian) acquired points is put into the tensor S.

The system only trains two tensors, H of size [RO,PE,NNeighbors, Nch, NTS] and M of size [RO,PE,NTS].

The regridded “segments (?)” are ${K}_{i,j,t}={\sum }_{n,c}{S}_{i,j,n,c}{H}_{i,j,n,c,t}$, and the reconstructed image is ${\hat{I}}_{i,j}={\sum }_{t}{K}_{i,j,t}{M}_{i,j,t}$.

While the 2nd multiplication is done pixel-wise in image-space, H is a non-Cartesian GRAPPA-like operation, i.e. a localized regridding kernel.

The generalization to multi-channel data is trivial and was implemented in the suggested networks.

## Supplementary information

Supplementary Information

## Data Availability

*Benchmark*: The benchmark reconstruction datasets generated during and/or analysed during the current study are available in https://github.com/giladddd/MLN/tree/master/Benchmark and the trained networks in https://figshare.com/s/65c0e9f77f23c664aabe. *Real data*: The reconstructed slices generated during during the current study are available at 10.6084/m9.figshare.7007774. The acquired subjects’ data for the current study are available from the corresponding author on reasonable request.

## Code availability

The code is available at https://github.com/giladddd/MLN. The training dataset used for the real data and benchmark test is a collection of randomly chosen slices from the HCP^38^ and can be accessed in the link above. For the real data, an acquired signal, the trajectory, NUFFT coefficients and time-segments data are included. For the benchmark test, the poisson-disc masks and the images used are provided, as well as the reconstructed images using various methods.
