# Supplementary material for: Minimal Linear Networks for Magnetic Resonance Image Reconstruction
Source: Sci Rep. 2019 Dec 20;9:19527. doi: 10.1038/s41598-019-55763-x (PMC6925115; doi:10.1038/s41598-019-55763-x)
Supplement: Supplementary file 1 — Supplementary Information [file 41598_2019_55763_MOESM1_ESM.pdf]

# Minimal Linear Networks for Magnetic Resonance Image Reconstruction

Gilad Liberman* and Benedikt A. Poser,
Faculty of Psychology and Neuroscience and Maastricht Brain Imaging Center
Maastricht University
The Netherlands

# Supplementary Material

## Figure S1

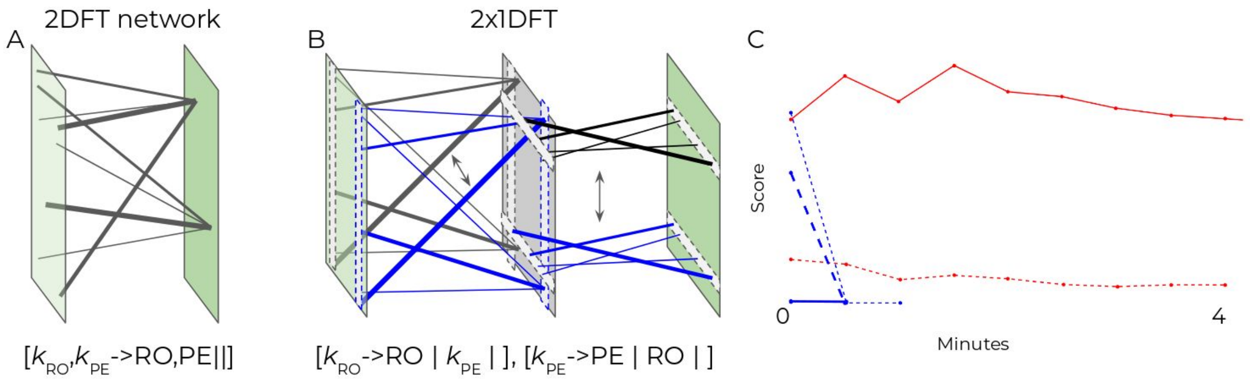


Fig. S1: 2DFT and 2x1DFT topologies. (A) 2DFT topology, a single FC layer. (B) 2x1D-FT topology. (C) Convergence graphs: Topology A in red, B in blue. Thick lines are with N=128, thin: N=64. For topology A only N=64 has fitted into memory. Solid lines: learning rate=0.002, Dashed: 0.0002.

# Figure S2

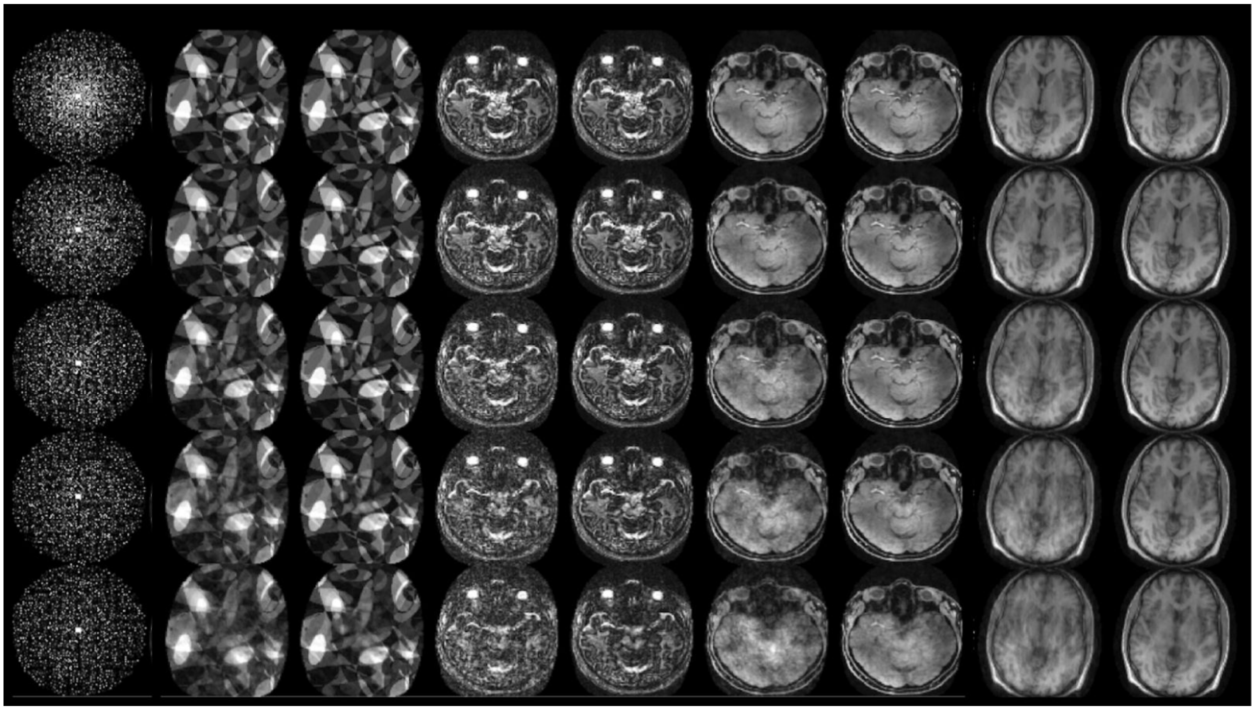

Fig. S2: Results of the reference method (BART, left) and the proposed method (right) at various undersampling factors and test images. The rightmost two images are complex valued, taken from real data (though not reconstructed directly from the signal).

# Table S1

SSIM scores for the various images and acceleration factors. ZF: Zero filling. DictL: Dictionary learning.

| Undersampling factor: | 8.8 | 11.1 | 12.9 | 14.9 | 17.5 |
|---|---|---|---|---|---|
| ZF, Brain | 0.778 | 0.695 | 0.657 | 0.637 | 0.621 |
| BART, Brain | 0.964 | 0.950 | 0.908 | 0.863 | 0.808 |
| DictL, Brain | 0.940 | 0.915 | 0.866 | 0.823 | 0.774 |
| MLN, Brain | **0.974** | **0.973** | **0.969** | **0.951** | **0.927** |
| ZF, Foot | 0.670 | 0.569 | 0.535 | 0.503 | 0.483 |
| BART, Foot | 0.943 | 0.905 | 0.855 | 0.802 | 0.741 |
| DictL, Foot | 0.895 | 0.822 | 0.771 | 0.712 | 0.650 |
| MLN, Foot | **0.948** | **0.943** | **0.932** | **0.896** | **0.844** |
| ZF, Abdominal | 0.792 | 0.673 | 0.623 | 0.594 | 0.570 |
| BART, Abdominal | **0.997** | 0.984 | 0.933 | 0.875 | 0.815 |
| DictL, Abdominal | 0.984 | 0.938 | 0.865 | 0.808 | 0.746 |

| | | | | | |
|---|---|---|---|---|---|
| MLN, Abdominal | 0.994 | **0.993** | **0.991** | **0.970** | **0.917** |
| ZF, Abdominal Ax | 0.635 | 0.513 | 0.469 | 0.434 | 0.414 |
| BART, Abdominal Ax | 0.960 | 0.912 | 0.836 | 0.759 | 0.690 |
| DictL, Abdominal Ax | 0.902 | 0.827 | 0.743 | 0.671 | 0.608 |
| MLN, Abdominal Ax | **0.976** | **0.967** | **0.949** | **0.908** | **0.840** |
| ZF, House | 0.707 | 0.627 | 0.580 | 0.556 | 0.533 |
| BART, House | **0.962** | 0.926 | 0.858 | 0.790 | 0.724 |
| DictL, House | 0.902 | 0.821 | 0.746 | 0.696 | 0.653 |
| MLN, House | 0.949 | **0.941** | **0.930** | **0.877** | **0.794** |
| ZF, Phantom | 0.701 | 0.620 | 0.575 | 0.557 | 0.537 |
| BART, Phantom | 0.940 | 0.918 | 0.862 | 0.806 | 0.748 |
| DictL, Phantom | 0.756 | 0.672 | 0.619 | 0.595 | 0.568 |
| MLN, Phantom | **0.945** | **0.945** | **0.935** | **0.904** | **0.851** |
| ZF, MP2RAGE Inv1 | 0.723 | 0.641 | 0.605 | 0.574 | 0.553 |
| BART, MP2RAGE Inv1 | 0.926 | 0.872 | 0.789 | 0.720 | 0.660 |
| DictL, MP2RAGE Inv1 | 0.893 | 0.842 | 0.764 | 0.698 | 0.641 |
| MLN, MP2RAGE Inv1 | **0.961** | **0.957** | **0.940** | **0.895** | **0.824** |
| ZF, MP2RAGE Inv2 | 0.696 | 0.609 | 0.569 | 0.545 | 0.523 |
| BART, MP2RAGE Inv2 | 0.953 | 0.913 | 0.842 | 0.756 | 0.688 |
| DictL, MP2RAGE Inv2 | 0.906 | 0.832 | 0.756 | 0.685 | 0.623 |
| MLN, MP2RAGE Inv2 | **0.973** | **0.969** | **0.959** | **0.926** | **0.872** |

# Figure S3

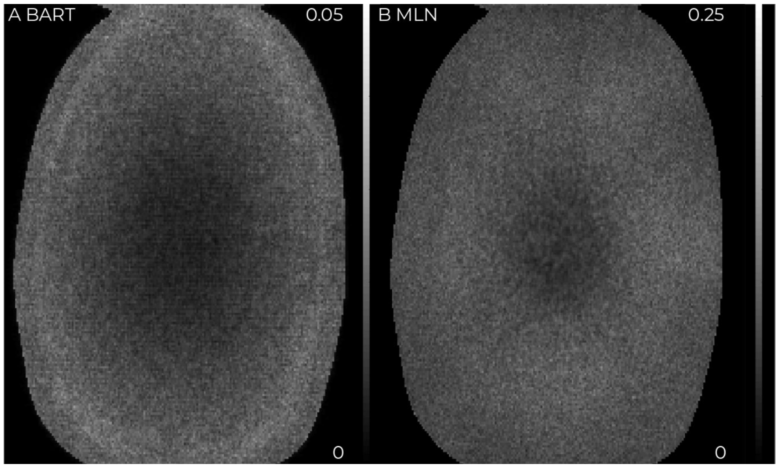


Fig. S3: Noise in simulated (Monte-Carlo) experiment.

# Figure S4

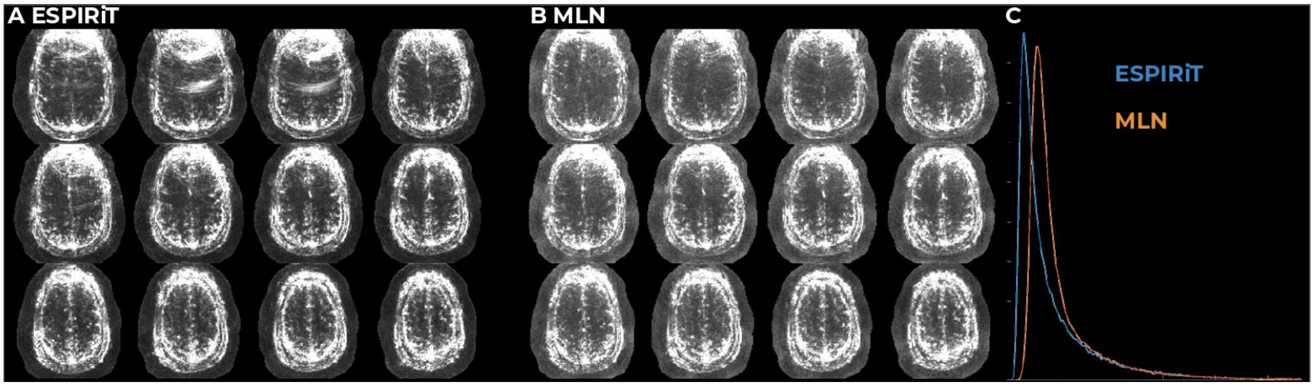


Fig. S4: Map of standard deviation over time, for complex multi-channel input data, observed in the reference ESPIRiT (A) and proposed MLN (B) reconstructions; (C) shows the corresponding histograms.

# Dataset 1

A : Summary of proposed and reference reconstruction methods on real data for all subjects can be found at https://doi.org/10.6084/m9.figshare.7007777

B : Same, detailed for every slice, at https://doi.org/10.6084/m9.figshare.7007774
